# Optical Density-Based Methods in Phage Biology: Titering, Lysis Timing, Host Range, and Phage-Resistance Evolution

**DOI:** 10.3390/v17121573

**Published:** 2025-11-30

**Authors:** Stephen T. Abedon

**Affiliations:** Department of Microbiology, The Ohio State University, Mansfield, OH 44906, USA; abedon.1@osu.edu

**Keywords:** bacteriophage therapy, latent period, lysis profile, MPN, phage organismal biology, phage therapy, resistance suppression

## Abstract

More than a century ago, bacteriophages (phages) were discovered as entities that could both replicate and dramatically reduce bacterial culture turbidities. By the late 1940s, phage impact on broth turbidity was being studied using electronic detectors. This review examines such turbidimetric, also known as colorimetric or optical density means of studying phage biology. The focus is especially on relatively rapid and higher throughput phenotypic phage characterization versus methods that rely instead on phage plaques, spots, or genotype determinations. Topics covered include (i) the most probable number method along with Appelmans’ approach, (ii) estimation of phage growth parameters including especially that of phage lysis timing, (iii) consideration of lysis inhibition as a complicating factor, (iv) phage titering based on degrees of optical density change, (v) detection of both lysis from without and resistance to lysis from without, (vi) phage host-range determination, and (vii) study of post-lysis culture grow back, that is, of bacterial evolution of phage resistance. Based on over 30 years of experience using and studying optical density approaches to the exploration of broth-culture phage biology, the author takes a critical look at both the benefits and limitations of this increasingly common approach to phage biological characterization.

## 1. Introduction

In 1922, phage co-discoverer [1] Felix d’Herelle described his “Fundamental experiment” [2] (p. 19): “The inoculation was sufficiently heavy to present a definite turbidity, but after incubation for twelve hours it was again clear.” John Douglas, in his 1975 monograph *Bacteriophages* [3], elegantly captured the mechanistic underpinnings of that observation (p. 14): “What one is witnessing is the progressive multiplication of the phage…” Here, I consider the utility of such turbidity-based analyses of phage-mediated clearing of broth bacterial cultures for characterizing various aspects of phage antibacterial biology.

Bacteriophages, or phages, are bacterial viruses. Their general life cycle consists of: (i) encounter with an adsorbable cell, (ii) attachment, (iii) infection, and then, for productive infections, (iv) a virion release step [4,5]. Two categories of phage release exist [6]. One can be dubbed as continuous, which involves virion extrusion [7] or, seemingly much more rarely, virion budding [8]; see also Baquero et al. [9] for archaeal virus continuous release. Alternatively, release occurs via phage-induced bacterial lysis [10,11]. This lysis is associated not just with the liberation of intracellularly produced virion particles but also with the physical destruction of the phage-infected cell. That destruction results in termination of cell-associated virion production as well as dramatic reductions in the ability of the now-lysed cells to scatter light. Phage-induced bacterial lysis results in substantial decreases in the turbidity of bacterial cultures, and these reductions occur just as virions are being released into the extracellular environment.

Detection of this lysis—such as by turbidimetric, also described as colorimetric or optical density means—can be used to characterize certain phage properties, such as lysis timing or phage host range. The approach can be useful as a labor-saving technology, including toward the development of phages as antibacterial treatments [12,13,14,15,16,17,18]. Historically, such assays were done using comparative, by-eye assessments of turbidity presence [19]. Subsequently, electronic means of optical density determination were developed [20]. In modern times, 96-well microtiter plates with automated measurement are typically used [21].

Changes in turbidity typically are quantified in the phage literature in terms of a culture’s optical density or OD, though some authors use equivalently, “Absorbance”. The wavelength of the light employed is often indicated in nanometers as a subscript, e.g., and most commonly used is OD_600_. Consistently, Storms et al. [22] recommend using wavelengths between 500 and 650 nm. Resulting decreases in the optical density, absorbance, or turbidity of bacterial cultures can be described as lysis profiles [23,24,25,26], though terms such as “Phage liquid culturing” [13] and “Liquid lysis assay” [27] have been used as well.

The traditional, non-optical means of following this process is the single- or one-step growth experiment [28,29,30,31,32,33]. One-step growth experiments can provide more information than lysis profiles, particularly that of phage burst size; they, however, can also require substantially greater effort. With one-step growth, the virion release step is characterized as a rise, meaning an increase in the infected culture’s phage titer. A key additional difference between lysis profiles and one-step growth experiments is that the latter is typically performed with relatively few of the bacteria being phage-infected, e.g., 10% or fewer. Lysis profiles, for the lysis step to be easily detected, by necessity require instead that a much larger fraction of the bacteria present be phage-infected. This higher infection level, however, is not necessarily required at the start of experiments.

This review examines various lysis profile-based approaches to the characterization of phage biology, organized by their primary applications. These include:

- Determining phage titers (Section 2 and Section 5);
- Measuring phage lysis timing (Section 3);
- Characterizing the phenomenon of lysis inhibition (Section 4);
- Detecting lysis from without as well as resistance to lysis from without (Section 6);
- Assessing phage ability to impact different bacterial strains (Section 7); and
- Gauging phage suppression of bacterial evolution of phage resistance (Section 8).

All involve the addition of some quantity of phages to a broth culture containing some concentration of planktonic bacterial cells. Each section addresses both methodology and critical limitations of these approaches.

Though often quite useful, shortcomings of these optical density-based approaches can include: (i) requirements for sufficient bacterial densities to easily detect culture turbidities, (ii) complications from use of endpoint rather than kinetic determinations, (iii) the potential for bacteria to evolve resistance to phages during assays, and (iv) limitations on phage abilities to effectively or rapidly lyse even cultures consisting of mostly phage-sensitive bacteria. Multiple other factors potentially impacting successful optical density-based characterizations of phage biology are also addressed.

The review begins with consideration of the most probable number method of phage titering. See Daubie et al. [34], Glonti and Pirnay [13], and Panteleev et al. [35] for much broader reviews of approaches to assaying the lytic activity of phages. See Table 1 for a summary of relevant terms.

## 2. Most Probable Number Method (MPN)

Plaque formation represents the current, typical means of viable phage quantification [13,29,36,37,38,39,40,41]. Not all phages or circumstances, however, are conducive to plaque formation, particularly using typical plaquing conditions [42]. At least three alternative methods of phage quantification have traditionally existed that rely on measures of phage-bacteria interaction. These are known as routine test dilution as used in phage typing [43], killing titer determination as used especially with phages that have been inactivated but not necessarily inactivated in terms of their ability to adsorb and kill bacteria [29,37,44,45,46,47,48,49], and the MPN method [37,49,50,51]. Only the latter is an optical density-based process, however, and that is what is considered in this section, along with the related technique by Appelmans (Section 2.4). See as well, though, Section 5 for an additional, also optical density-based approach to phage titering. Discussed in the current section also is the utility of kinetic vs. endpoint turbidity determinations (Section 2.3).

### 2.1. MPN Method

Krueger [19] indicated, in 1930, p. 557, that “Two general methods for the quantitative determination of bacteriophage are known.” These are plaquing and “testing serial dilutions of the lytic agent for ability to produce complete dissolution of a broth culture of susceptible bacteria.” Ideally, one then employs statistics to infer starting phage titers from such broth culture dissolution, i.e., as it can occur within multiple broth tubes containing both phage dilutions and susceptible bacteria. This approach has been described, at least since 1933 in the phage literature, as the most probable numbers method [51]. Benzer et al. [44], however, described the general technique instead as “Titration by serial dilution to the limit of activity” while Douglas [3] used “Dilution to extinction”.

The MPN approach explicitly involves optical density determinations. These determinations, though, do not necessarily require a quantified measure of optical density. Rather, culture turbidity can instead be assessed by eye, as has been done traditionally. Its basis, for phages, is first the potential for a single phage virion to modify a bacterial culture over time, explicitly preventing the formation of noticeable turbidity, and second, the ability of a single bacterium to generate a noticeably turbid culture in the absence of infecting phages. The goal in either case is to infer original titers based on what dilution is sufficient to result in less than all inoculated tubes displaying turbidity, e.g., see the second figure of Geng et al. [52], panel e. An assumption of a Poisson distribution—generally provided as published tables—is then employed to calculate pre-dilution phage titers [51]. Here, a phage-positive reaction would be culture clearing. That contrasts with when assaying for bacterial numbers using the MPN method, where the bacteria-positive reaction instead would be turbidity presence.

The MPN method, thus, is based on individual phages giving rise to countable tubes that indicate phage presence in the form of a lack of prominent culture turbidity. Plaque counting similarly is based on individual phages giving rise to discrete countable units, each also indicating phage presence. The two approaches are consequently both about counting where phages have locally inhibited the turbidity of a bacterial culture. In either case, utility is dependent on that inhibition being consistently obvious.

### 2.2. MPN Cautions

A concern when using MPN-type assays to determine phage titers is that starting bacterial concentrations need to be appropriate to allow a single phage to give rise to culture-wide bacterial lysis. This is the concept of limitations to phage antibacterial virulence [2,22,49,53,54,55,56,57]. These limitations stem from not just phages but also bacteria replicating in the course of these assays. Specifically, phages can increase their population sizes only so fast, and that increase and/or phage-induced culture-wide bacterial lysis typically is inhibited if bacterial cultures are allowed to approach stationary phase. Consequently, if starting bacterial densities are too high (e.g., 10^8^ bacteria/mL or higher) or culture volumes are too large (e.g., multiple milliliters rather than fractions of milliliters), then sufficient culture-wide bacterial lysis will not occur despite the presence of a single starting phage virion, even if that phage can productively infect the bacteria present. The result can be an underestimation of phage titers using this method, an issue that is explicitly indicated by Carlson [37].

Similarly, Adams [29] notes in describing issues with “Dilution end-points”, p. 30, that

*…if the adsorption is poor or phage multiplication slow, lysis may occur only when a considerable number of phage particles are inoculated, and so the phage population will be grossly underestimated. In such cases, the estimate can be improved by testing for presence of phage in all tubes in which lysis failed.*

If a single phage is thus unable to lyse a bacteria-seeded broth tube, then there is a need to start with fewer bacteria. It is inconvenient, however, to check for phage presence in all unlysed tubes. Consequently, the possibility that it may require more than one starting phage to result in complete lysis of a culture should be first explored if relying on MPN methods for phage titering. See Section 2.4 for additional consideration of this concern.

Another MPN limitation, as is the case with endpoint optical density-based approaches generally, is the potential for regrowth of cultures by phage-resistant bacteria [13] (Section 8). This can result in substantial endpoint culture turbidity despite the presence of one or more phages having successfully prompted culture-wide bacterial lysis. There, therefore, exist three possible broth culture optical density endpoints given the presence of both phages and bacteria:

- Minimal turbidity indicates phage-induced culture-wide bacterial lysis;
- Failure of a culture to lyse despite phage presence (a false negative due to insufficient phage antibacterial virulence, but see also Section 4); and
- Occurrence of culture-wide bacterial lysis that is followed by growth of phage-resistant bacterial mutants (also a false negative result; Section 8).

Note, though, that it is possible to mitigate especially this latter issue by using kinetic rather than endpoint analyses.

### 2.3. Kinetic vs. Endpoint Analysis

To detect culture-wide bacterial lysis using turbidimetric means, it is necessary to take measurements at time points that both follow phage-induced, culture-wide bacterial lysis and are prior to any grow back of phage-resistant bacteria [55] (also known as “regrowth”; Section 8). Kinetic determinations, unlike endpoint determinations, are not limited to one specific datum and therefore do not need to correspond solely to such optimal time points. Consequently, kinetic analyses can be more reliably interpreted as indicators of the occurrence of culture-wide, phage-induced bacterial lysis than endpoint determinations. Note, though, that it is also possible for kinetic analyses to fail to detect substantial phage-induced bacterial lysis, and this can be particularly problematic if assays are terminated too soon.

Though in some cases endpoint determinations certainly may work, for the rest of this review, save for the following section (Section 2.4), it is assumed that kinetic determinations of changes in bacterial culture optical density will be used. Such kinetic analyses are either manually determined, e.g., by using a Klett-Summerson Photoelectric Colorimeter—see Adams [58] and Baer and Krueger [59] for what may be the first and second phage lysis profile generation using “Klett readings”—or instead can be obtained in a more automated fashion [21].

### 2.4. Appelmans’ Method

Similar, but less quantitatively sophisticated than the MPN method, is the earlier (1921) method of Appelmans [60]. This uses the highest dilution, for which phage-induced bacterial lysis still occurs, to calculate phage titers [13,60]. Niu et al. [54,61,62] seem to describe the same sort of protocol as defining a phage’s “lytic capability”, which they label also as a “virulence assay”. Appelmans’ method can also be described as an endpoint dilution. It in addition can be viewed as equivalent to a routine test dilution, but with the assay involving broth cultures rather than agar plates.

A protocol for Appelmans’ method is provided by Chanishvili [63], pp. 265–266, with “log” added to clarify:

*A serial (10-fold sequential) dilution of a phage stock is required. …bacterial suspension should be added to all ten tubes … Evaluation of the results should be performed by comparing transparency of all the 12 tubes in the row. Culture control should become turbid demonstrating growth of the bacteria, while the media control should remain transparent, demonstrating sterility of the tubes and their content. The titer of test bacteriophage is estimated by the last dilution which remains transparent (this means that lyses of the bacterial suspension in this tube still occurs). The titer determined by Appelmans [60] is expressed by negative [log] figures corresponding to the dilution.*

Chanishvili goes on to note (p. 266) that (emphasis mine), “*Appelmans[‘] method allows only an approximate estimation of the phage titer*, while the method [based on plaque formation] is much more precise.” This difference in part will be due to the reliance of Appelmans’ on simply powers-of-ten differences, though obviously smaller dilution increments can be used instead. The issues raised in Section 2.2 regarding phage virulence, however, may help to explain additional discrepancies between Appelmans-based titering and that based on plaquing. Note also that, as documented by Chanishvili [63], p. 18, Appelmans-based titers can decline as those assays are extended, e.g., from 6 h to 24 h, which could be due to regrowth of phage-resistant bacteria, thereby converting phage-positive dilutions to phage-negative dilutions over time (see again Section 2.2).

An additional consideration is that Appelmans’ method of phage titering is somewhat distinct from the Appelmans protocol used to enhance phage antibacterial properties for phage therapy [13,34,64,65,66,67] (for completeness, though not explicitly mentioning Appelmans’, see also [68]). In my reading of Appelmans [60], in translation [69], there however is no mention of evolving bacteriophages. Instead, the article considers the quantitative determination of the impact of various substances on phage virion viability (alcohol and carbonic acid). To avoid making false claims about the contributions of Appelmans, a systematic search was undertaken for additional Appelmans publications, with a total of three further Appelmans publications identified. Two of these, however, are not single-authored and so would not be technically just Appelmans’ [70,71,72], and the third has been translated into English, allowing confirmation by this author that it is not a phage-evolution study [73].

### 2.5. Consideration of Bacterial Initial Physiological State

It is both common and convenient to initiate phage experiments directly using diluted overnight cultures, e.g., [74,75,76]. One issue with this approach is that not all overnights are identical, varying in terms of how long they have been incubated prior to storage as well as how long they have been subsequently stored prior to use. This practice of using overnight cultures for experiments, even if first diluted, can also be expected to modify phage life-history characteristics such as latent period length, burst size, and virion adsorption rates. Together, these modifications of phage growth parameters can slow initial rounds of phage population growth. For MPN-type experiments, it can also be preferable to start with bacterial physiological states that are most likely to minimize single-phage losses upon their initial adsorption step, such as by employing mid-log phase bacteria for assays.

Carlson [37], p. 442, provided suggestions as to how to go about doing this:

*Starter cultures may be initiated with a single colony and grown overnight or longer under whatever conditions the bacteria favor. Such cultures normally enter stationary phase, and after dilution they will lag for an irreproducible period of time before resuming growth. Therefore, they need to be diluted at least 100-fold and go through several divisions to ensure that all cells are in the same growth phase at the time of infection. An alternative approach works well with bacteria that do not lose viability upon rapid chilling: a starter culture grown to mid-log phase is immediately chilled in an ice bath and kept cold. Such cultures need only a 20-fold dilution for regrowth the next day, and the cells usually resume growth in a more reproducible manner than when stationary starter cultures are used.*

For the sake of temporal consistency and physiological reproducibility, experiments should thus be initiated with bacteria that have been grown to a mid-log phase physiology, unless it is phage infection of other bacterial physiological states that are being studied.

## 3. Estimation of Phage Life-History Characteristics

The genetics of phages and the biochemistry of phage infections give rise to what can be described variously as phage growth parameters, organismal-level phenotypes, or life-history characteristics [5,75,77]. These include especially virion adsorption rates, phage latent period lengths, and phage burst sizes. The most obviously obtainable of such information from optical density-based approaches is latent period length [78]. More broadly, this is the timing of phage-induced bacterial lysis, also described as lysis from within [79]. Such latent period determinations using lysis profiles typically will take less time, require less labor, and involve fewer consumables than the plaque-based one-step growth experiments. A recommended workflow should consist of: starting with sufficient numbers of metabolizing bacteria that changes in turbidity are easily detected (such as 10^8^ bacteria/mL) → addition of phages at a multiplicity of ~5 → monitoring the culture’s optical density kinetically for signs, especially of the initiation of lysis (turbidity reduction).

### 3.1. Inferring Lysis Timing Information

Phage “purely lytic” cycles [80] begin with virion adsorption and end with virion release, the latter corresponding to bacterial lysis. Alternatively, phages exhibiting a lysogenic cycle [81] can display “induced lytic cycles” [82]. In either case, the duration of a lytic cycle is usually well controlled by most lytic phages, thereby giving rise to characteristic timings of phage-induced bacterial lysis [10,11]. This lysis timing, nonetheless, can vary not just between phage types and genotypes but also as a function of the genotype of the hosting bacterium, or with physical and chemical environmental variation.

The key to determining latent period lengths using optical density measurements is a synchronization of the start of infections, thereby allowing for a degree of synchronization also of the termination of infections across a phage population. This is just as is the case with one-step growth assays, which instead employ plaque counts to assess the timing of phage lysis [29,33]. With purely lytic cycles, this requires that a majority of phage adsorptions occur over short periods of time. Unlike one-step growth experiments, however, for latent period determination using optical density means, a majority of bacteria must become infected during the initial adsorption period to generate a useful lysis profile. This more or less culture-wide phage infection of bacteria can be assured by employing a higher multiplicity of infection (>1) along with a relatively high bacterial concentration (e.g., 10^8^ bacteria/mL; see Section 3.3 for further discussion of phage multiplicities). That higher-multiplicity approach, though, is not necessarily without complication (Section 4). For one-step growth, by contrast, typically a low multiplicity is desired (<<1).

**Figure 1:**
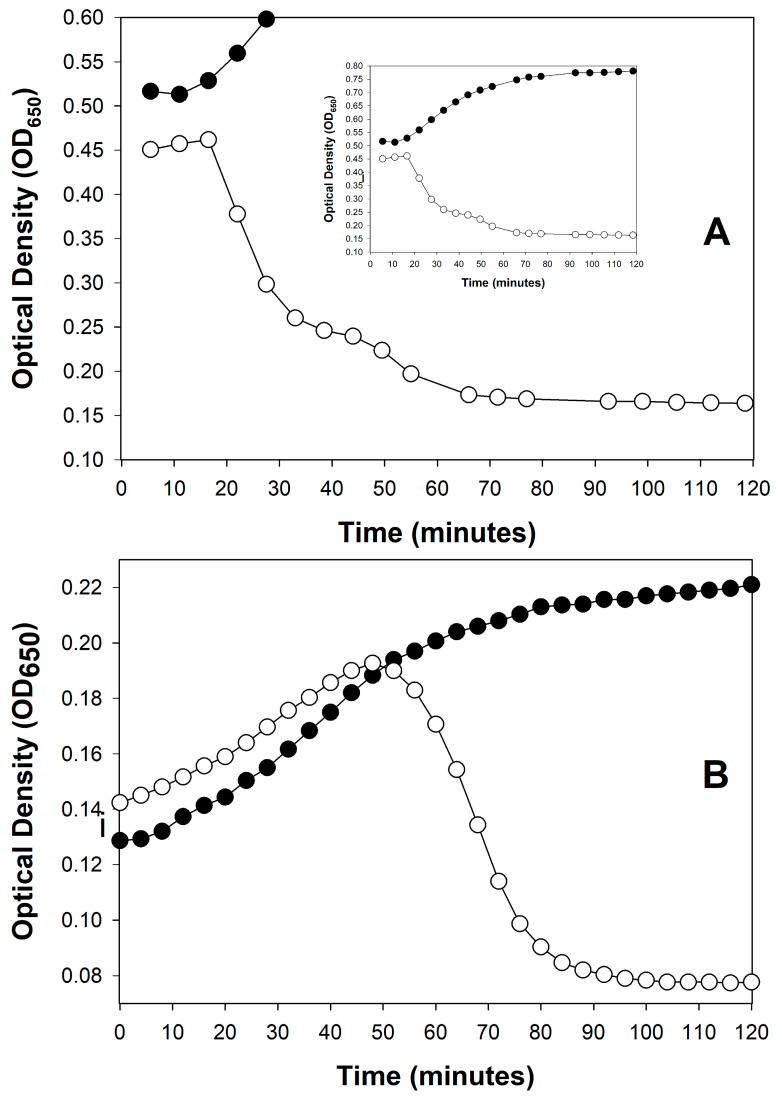
Examples of sudden, phage-induced, culture-wide bacterial lysis by different phages under different experimental conditions. Curves are distinguished into phage-free bacteria (closed circles, ●) and bacteria plus added phages (open circles, ○). (**A**): Shown is a purely lytic infection cycle of phage T4, starting with 2.5 × 10^8^ bacteria/mL and 5 × 10^8^ bacteriophages/mL, as based on the Supplemental data supplied by Rajnovic et al. [76]. The experiment is suggestive of a latent period of ~20 min, which is roughly as expected [84]. This is indicative of the utility of optical density-based methods toward at least ballparking phage latent period lengths before moving on to the more labor-intensive one-step growth experiments. Importantly, notice as well the relative lack of display of lysis inhibition (Section 4), which presumably is a consequence of the use of a starting phage multiplicity of only 2 in this experiment. A slight amount of lysis inhibition may actually be occurring, however, visible as the slight slowing in turbidity decline visible starting around 40 min. I speculate, however, that this did not affect the initial timing of the start of lysis, i.e., as determines the end of the constant period (as latent period length). (**B**): Shown is the result of a series of purely lytic cycles by phage T3 that are due to starting with a phage multiplicity of somewhat less than 1. At the point of approximately peak turbidity in the phage-containing culture, phage infection of the majority of bacteria present has commenced (the peak is seen in the graph at 45 to 50 min). Soon after—as the latent period of phage T3 is only about 13 min in length [84]—phage-induced culture-wide bacterial lysis ensued. See Appendix A for protocol details.

For induced lytic cycles, an efficient inducing agent must instead be supplied to the lysogenized bacteria so that lytic cycles in a majority of bacteria are induced, also over a short period. This can be done, for example, by using a thermally inducible phage λ mutant [24,83]. The end of a latent period—regardless of its means of initiation—ideally will be observed, optically, as a somewhat rapid drop in turbidity, indicating culture-wide bacterial lysis (Figure 1).

Figure 1 shows two examples of such somewhat rapid drops in culture turbidity, one following only a single round of phage infection (Figure 1A) and the other following multiple rounds of phage infection (Figure 1B). Only the former, starting with a phage multiplicity of greater than one, may be used to directly infer phage latent period lengths. See Danis-Wlodarczyk et al. [85] for a more in-depth comparison of single- and multiple-step optical density-based determinations of lysis timing. Keep in mind in any case that the precision of lysis timing determinations will depend on the length of intervals between time points, as also is the case with one-step growth experiments [33]. Differences between phages in the range of a few minutes generally can be relatively easily distinguished, so long as sufficient numbers of time points are taken.

### 3.2. Inferring Other Phage Life-History Information

Turner et al. [75] is a methods paper. There, they combine measures of phage impact on bacterial culture turbidity—starting with less-than-one phage multiplicities—and mathematical modeling of phage population growth to infer phage organismal phenotypes beyond just lysis timing. From this, they describe “growth-associated virus traits”. See similarly the work of Geng et al. [52] and Blazanin et al. [77]. The approach differs from determinations of strictly lysis timing, where instead multiplicities of greater than 1 should be employed (Section 3.3). Overall, the Turner et al. effort represents a promising approach, especially toward estimating how differing phage life-history characteristics may individually impact phage evolutionary fitness during broth-culture growth, or toward the development of phage-choice criteria for phage therapy purposes [77].

### 3.3. Multiplicity of Infection During Optical Density-Based Latent Period Determination

By multiplicity of infection as used above, what is implied is the ratio of adsorbed phages to phage-susceptible bacteria, which can be described instead as a multiplicity of adsorption. This distinction is made because, due to phages displaying superinfection exclusion [86,87,88], not all adsorbing phages necessarily successfully infect. More importantly, however, is the distinction between the ratio of phages added to bacteria versus the ratio of adsorbing or infecting phages—MOI_input_ vs. MOI_actual_ [89]. Specifically, if phages are slow to adsorb, then MOI_input_ can be a poor predictor of MOI_actual_. A further issue is that if bacterial concentrations are low, then for a given MOI_input_, phage titers will also be low. Thus, especially when working with phages that are intrinsically slow to adsorb, MOI_input_ can be a poor predictor of MOI_actual_ [90].

Assuming one is working with a legitimate MOI_actual_, the goal when determining latent period lengths—using optical density means—should be to infect a sufficiently large fraction of bacteria that the timing of their lysis is easily inferred. This fraction can be calculated as 1 − e^−MOI^, based on assumptions of a Poisson distribution, with “MOI” there equal to MOI_actual_. With an MOI_actual_ equal to 5, the expectation is that 99% of cells will be phage-infected. Alternatively, with an MOI_actual_ of just 2, the expectation is that 86% of bacteria will be phage-infected, which should be sufficient to infer when lysis is occurring. Thus, if starting with an MOI_input_ of 5, then even with slow-adsorbing phages an MOI_actual_ of 2 may still be achievable. If not, one can start with a higher MOI_input_.

An objection to the latter suggestion could come from concerns over the potential for lysis from without (Section 6). However, there is both a workaround for that concern and a reason to not be otherwise concerned. The workaround involves simply testing different values of MOI_input_, such as 2, 5, and 10 [85]. If one observes substantially sooner lysis with the higher ratios, then lysis from without can be suspected. Lysis from without nonetheless is likely not a concern because phages that can give rise to lysis from without also are expected to give rise to resistance to lysis from without (Section 6.1). Since determining phage latent period lengths requires working with metabolizing bacteria—in most cases, bacteria in mid-log phase of growth—one should expect that resistance to lysis from without will be indeed expressed by initially adsorbing phages. Also, traditionally lysis from without is not expected unless MOI_actual_ is much higher than needed for optical density-based latent period determinations, e.g., 100 rather than 5.

Notwithstanding these various approaches to overcoming multiplicity-related issues, use of optical density-based approaches to lysis timing or other phage growth parameter determinations can still be complicated by the existence of a phenomenon seen with certain phages, called lysis inhibition.

## 4. Lysis Inhibition as a Lysis Profile Complicating Factor

**Figure 2:**
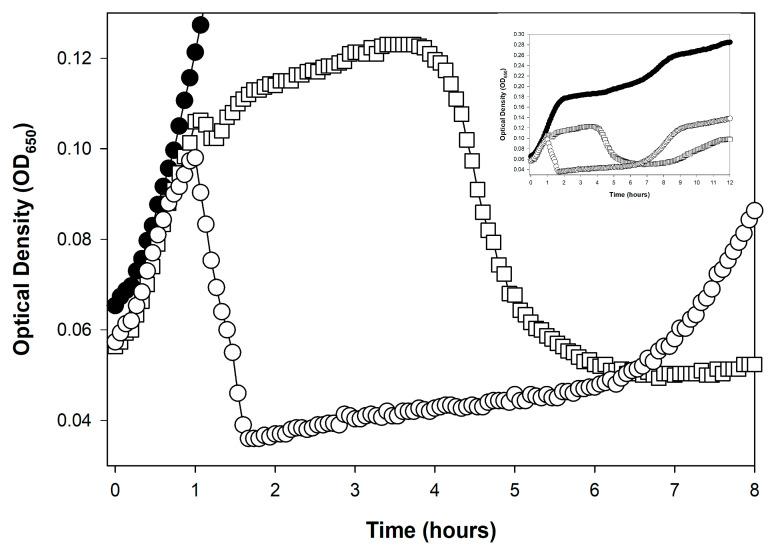
Rapid lysis vs. lysis inhibition. Rapid lysis is the term that can be used to describe a non-lysis-inhibited lytic cycle [91]. It is seen here associated with a phage T4 *r* mutant (*r* for “rapid lysis”). Compare the lysis profile associated with phage T4 wild type (T4D, open squares, ☐), which avoids a dramatic lysis event until roughly four hours after phage application, with roughly one hour for the mutant (T4 *r67*, a gene *rIII* mutant, open circles, ○); the closed circles curve (●) is phage-uninfected *E. coli* B. The early dip in the T4D curve, between 1 and 1.5 h, is presumably indicative of the first round of phage-induced bacterial lysis. That lysis should generate the secondarily adsorbing phages responsible for inducing lysis inhibition in the remainder of the phage-infected bacteria (here starting around 1.5 h and ending between roughly 4 and 5 h). Also for phage T4D, the subsequent rise in turbidity, starting around 1.5 h in the figure, has been found to be associated with an increase in the size of individual bacteria rather than being due to ongoing bacterial division [109,110]. The drop in turbidity, starting for T4D around hour 4, is associated with phage-induced bacterial lysis and can be dubbed a lysis-inhibition collapse [111]. The drop in turbidity of around 1 h with the *r67* mutant, by contrast, is simply lysis, or instead what may be dubbed as the noted rapid lysis [92]. The late increase in turbidity, especially starting around 7 h for the *r67* curve (○), is assumed to be associated with the growth of phage-resistant bacterial mutants (Section 8). The equivalent regrowth is delayed (and not seen in the figure) in association with the phage T4D infection ☐), perhaps due to competition for oxygen between phage-resistant bacteria and the lysis-inhibited bacteria. This experiment otherwise was conducted equivalently to that shown in Figure 1B, except representative individual technical repeats are shown rather than averages of three technical repeats.

An obstacle to precise optical density-based determinations of phage latent period lengths can result from a phenotype known as lysis inhibition [20,49,91,92,93,94,95,96,97,98,99,100,101,102,103,104,105,106] (and see also [107]). Lysis inhibition is an extension of the phage latent period, and particularly it is an extension that is induced by the adsorption of an already phage-infected bacterium by a phage of equivalent type (secondary adsorption). For example, this can be the adsorption of a coliphage T4 virion to an already coliphage T4-infected bacterium. Therefore, if a lysis profile experiment is initiated with phage multiplicities of somewhat greater than 1, then that can result in a substantial latent period extension, of up to many hours (Figure 2), though these extensions do not always occur (Figure 1A). The result of lysis inhibition is not only a delay in phage-induced bacterial lysis, but also an increase in the resulting phage burst size. Generally, that is, the longer the time between the start of a lytic cycle and subsequent lysis, all else held constant [108], then the larger the average phage burst size.

Workflows for lysis-inhibition assays will vary due to a number of seemingly poorly defined variables, and are also expected to vary between phage types and bacterial hosts. As a general rule, the goal should be to set up the experiment to avoid excessively high peak concentrations of phage-uninfected bacteria, e.g., not too much greater than 10^8^ bacteria/ml, since phage latent periods can become extended due solely to infection of bacteria at high turbidities [21,22,49,52,55,74,76,112,113,114,115,116,117]. I have found that starting with a phage multiplicity of less than 1, such as 0.1 or lower, and then letting phage population growth result in sufficient phage multiplicities often results in robust lysis inhibition. For more controlled experiments, one can apply a multiplicity of phages of approximately 5, which is followed at some point with a second multiplicity of phages also of around 5, starting with 10^8^ bacteria/mL. In practice, though, varying conditions can be helpful for optimizing phage display of lysis inhibition, and ideally this will be done while ensuring that cultures don’t progress past mid-log phase before full phage infection is achieved. Discussed in this section also are strategies for avoiding phage display of lysis inhibition (Section 4.4).

### 4.1. What Is Lysis Inhibition?

Lysis inhibition is a phenotype associated particularly with obligately lytic phages and thus with purely lytic cycles of infection. Display of the phenotype is complex, requiring wild-type versions of at least seven different phage loci. The phenomenon has been most thoroughly studied with phage T4. There, mutations in separate loci that lead to defects in the phenotype are described as *rI*, *rIIA*, *rIIB*, *rIII*, *rIV* (also known as *spackle*), *rV* (which are alleles of gene *t*), and also gene *imm* (for *immunity*). An additional phage T4 gene, *5*, can also possess at least one mutation that can lead to a partially lysis inhibition-defective phenotype, in this case due to bypassing the role of the *spackle* gene [25]. Expression of the associated genes by initially adsorbing phages is required prior to either the first secondary adsorption or adsorptions that induce the lysis inhibition phenotype [97,98,99,100,101,102,106] or prior to excessive subsequent secondary phage adsorptions [97]. The signal supplied by the initially secondarily adsorbing phages is thought to be secondary phage genomic DNA [118].

In multistep phage population growth, where experiments are initiated with phage multiplicities of somewhat less than 1, phage secondary adsorptions become likely only once phage titers have caught up numerically with bacterial numbers. As a consequence, phages are expected to replicate first with little display of lysis inhibition, but then the culture, once phage virions are sufficiently numerous, will rapidly transition to most phage-infected bacteria displaying lysis inhibition, along with most susceptible bacteria being phage-infected. Phages unable to display lysis inhibition either possess mutations in lysis-inhibition-required genes, such as in the case of phage T4 described above, or lack entirely the genes required to display this phenotype (Section 4.3).

Display of lysis inhibition generally results in substantial delays in phage-induced bacterial lysis. This can range from less than 10-min increases in latent period length [119] to increasing that latent period manyfold, such as tenfold longer or even longer depending on the phage [95]. For example, latent periods can extend from an expected 20 to 25 min in length [84] to instead six or more hours long. These being purely lytic infections by obligately lytic phages, the expectation is that infections will end in an observable lysis [20,93], dubbed lysis-inhibition collapse [120].

### 4.2. Earlier vs. Later Lysis-Inhibition Induction

Because lysis inhibition is induced by the adsorption of one or more virions to an already phage-infected bacterium, it can represent a complication on determinations of phage lysis timing using optical density means (Section 3). This occurs in part because it can be difficult to simultaneously infect bacteria with higher multiplicities of phages—as required to infect a majority of bacteria present (Section 3.3)—while also avoiding secondary adsorptions of those bacteria (Section 4.3, though see the possible, perhaps partial workaround presented as Figure 1A and in Section 4.4.3). During one-step growth, by contrast, secondary adsorption is avoided at least initially by instead starting phage infections with low multiplicities of phages, i.e., since one-step growth does not require infection of a majority of bacteria present.

Secondary adsorption can occur not only during the initial period of phage adsorption and infection, it can also occur after lysis of some phage-infected bacteria has begun, which is later during phage infections (between approximately 1 and 1.5 h in Figure 2). In both cases, the result nonetheless is initiation of lysis inhibition. For the latter, it is phages that have been released from bacteria that have not yet been secondarily adsorbed that go on to adsorb not yet lysed phage-infected bacteria.

The resulting, induced lysis delay (or further-extended delays [119]) is thus initiated somewhat late in what otherwise would have been a normal lytic cycle. This explicitly is what is presumed to be seen in the wild-type (T4D) phage profile presented in Figure 2, since that experiment was initiated using somewhat low phage multiplicities (see also the slight delay seen during lysis in Figure 1A around 45 min). As there is a potential for phage secondary adsorptions to induce lysis inhibition both early and later in phage latent periods, precautions must be taken throughout optical density-based latent-period determinations to avoid lysis inhibition if these determinations are being used as an alternative to performing one-step experiments.

### 4.3. Not an Issue for All Phages

These precautions—to avoid inadvertent phage secondary adsorptions during lysis timing determinations—need only be taken when working with phages that are able to display lysis inhibition. Of the original “Type” coliphages, T1 through T7 [121], phages T2, T4, and T6, the so-called T-even phages [93,122,123], all display lysis inhibition while phages T1, T3, and T7 do not [91]. For wild-type phage T5, whether it displays lysis inhibition is ambiguous [20,93,124]. Based on the data presented in those articles, as well as preliminary experiments done in-house, my impression is that wild-type phage T5 probably does not display an equivalent secondary adsorption-induced latent period extension. For reports of lysis inhibition observed in additional phages, see [94,96,103,105].

### 4.4. Preventing the Lysis Inhibition Complication

If working with phages that can display the lysis inhibition phenotype, then precautions need to be in place if lysis profiles are to be used to determine phage latent-period lengths. This requires limiting phage secondary adsorptions throughout the phage lytic cycle, though practically speaking, this is most important near the start of infections and prior to normal (not lysis-inhibited) lysis from within (Section 4.1).

#### 4.4.1. Preventing Lysis Inhibition Early

One approach to avoiding secondary adsorptions near the start of infections is adsorption synchronization, as can be achieved by various means [35]. For instance, one can start with bacteria that are metabolically starved, e.g., by adsorbing cells that have first been washed in buffer, with concentrated media added or dilution into fresh media made only after adsorption has mostly gone to completion [125]. This prevents expression, over the course of the adsorption step, of phage genes that are necessary for display of lysis inhibition [98,100]. So too such gene expression can be avoided by employing metabolic poisons such as KCN, but then these inhibitors would need to be effectively removed, such as via centrifugation and subsequent cell resuspension in fresh media [125].

Alternatively, faster adsorption may be achieved by starting with mid-log phase bacteria that have been concentrated, such as to 10^9^ cells/mL, and with the phage-bacteria mixtures then diluted down to, e.g., 10^8^ cells/mL within fresh media in the course of reinitiating metabolism. The latter can also include diluting into media that is less ideal for supporting phage adsorption, such as into media missing necessary adsorption cofactors [4]. These approaches, however, all have the potential to modify bacterial and thereby infection physiologies. Such strategies nonetheless should allow for relatively easy comparative lysis timing determinations so long as the same protocol is consistently employed.

#### 4.4.2. Preventing Lysis Inhibition Later

Alternatively, one can inactivate potential secondarily adsorbing phages both at the beginning and toward the end of normal latent periods by adding anti-phage serum. This can work to prevent subsequent secondary adsorptions even if added relatively late in infections [120]. Use of antiserum to prevent further phage adsorptions is an approach that has also been used for one-step growth experiments to better synchronize phage adsorptions [29]. Chemical virucides potentially can serve a similar anti-adsorption function as anti-phage serum (as listed in Abedon et al. [126]).

Another approach to avoiding later secondary adsorptions, though one that is more limited in its applicability, is to employ phages that upon release are adsorption-defective. An example is the use of phage T4 gene *37* amber mutants experimentally infecting a non-amber suppressing host [127]; gene *37* encodes the distal portion of phage T4 long tail fibers, i.e., the part that first interacts with the surface of the host cell during adsorption [128,129,130]. With one-step growth experiments, by contrast, lysis inhibition is avoided later in experiments by diluting cultures so as to reduce the concentrations of virions released [33].

#### 4.4.3. Easier Approach That Needs More Testing

Without any of these suggested workarounds, Rajnovic et al. [76] nonetheless appear to have succeeded in mostly avoiding lysis inhibition in an optical density assay using phage T4. As presented here in Figure 1A, this was done while employing a somewhat high bacterial density of 2.5 × 10^8^ bacteria/mL in combination with a multiplicity of 2—virions therefore should have adsorbed to near completion very rapidly. One can see a similar result in the same study starting instead with 5 × 10^8^ bacteria/mL (multiplicity of 1) or with only 10^8^ bacteria/mL (multiplicity of 5), though at the latter cell density, the lysis may have been slightly more gradual, suggesting some lysis inhibition. These results nonetheless all point to the possibility that, by varying both phage multiplicities and starting bacterial concentrations, expression of lysis inhibition may be reduced enough to allow latent period-length determinations. The goal—however it is attained—is for lysis inhibition to be sufficiently uncommon in the course of an experiment that, despite a given phage’s propensity to display that phenotype, lysis timing may still be easily inferred from lysis profile data. That is, even if fewer than 100% of bacteria participate in the initial culture-wide bacterial lysis, the timing of the start of lysis can still be discernible.

Note that difficulties in avoiding phage display of lysis inhibition can similarly be a concern during kinetic optical density-based approaches to assessing phage virulence [22] (Section 7.2.4). An equivalent issue can be seen with efforts to employ kinetic optical density measurements to estimate phage titers (next section).

## 5. Titering Phages Based on Kinetic Optical Density Measurements

**Figure 3:**
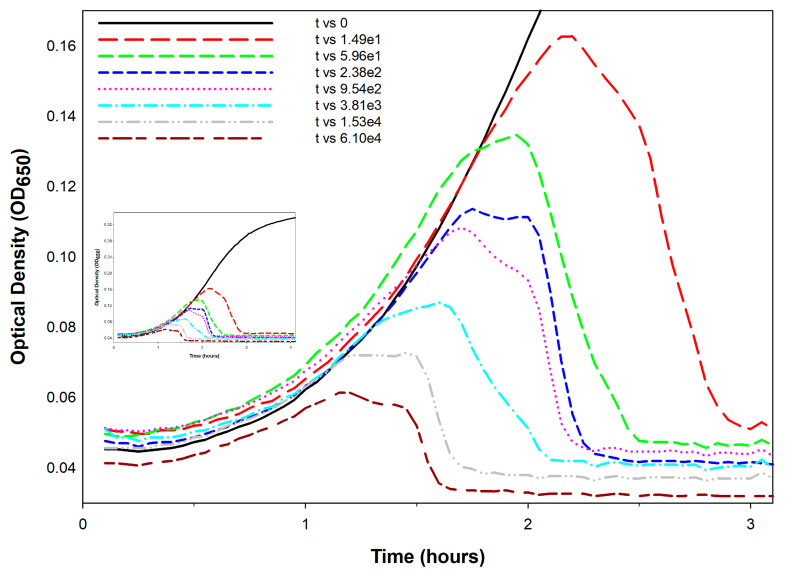
Optical density-based phage titer estimation assay. Shown is an example of how lysis profiles can vary as a function of starting phage titers, here employing a lysis-inhibition defective phage T4 *r48* mutant infecting *E. coli* B. The bacteria were grown at 37 °C in trypticase soy broth (TSB) supplemented with 2.9 g/L NaCl. Starting titers in this experiment varied eight-fold between individual curves as indicated in the legend (phages/mL). See Appendix A for additional experimental methods.

First developed, to the best of my knowledge, in 1930 by Krueger [19] (and cited for that as recently as 2018 [131]), it is possible to use kinetic optical density assays to estimate the concentration of phages added at the start of lysis profile experiments. This approach has been further developed recently by Rajnovic et al. [76] and then by Geng et al. [52], but see also Maillard et al. [74]. I subsequently have reviewed these efforts in some detail [49], dubbing them there as “KOTE” assays (for Kinetic Optical density-based Titer Estimation). The resulting experiments otherwise resemble the noted phage antibacterial virulence assays [2,22,49,53,54,55,56,57]. See Figure 3 for an illustration of what the resulting starting-titer-differentiated curves can look like.

The underlying principle of KOTE assays is that, when starting with sufficiently low phage numbers—low enough that it takes multiple rounds of phage infection and lysis to infect a majority of bacteria present—then there can be substantial delays in the timing of subsequent phage-induced bacterial lysis. These delays should be longer the lower the starting phage titer. Furthermore, the extent of these delays can be reasonably consistent from experiment to experiment, thereby opening up the possibility that their kinetics can be used to estimate starting phage titers. The timing of phage-induced bacterial lysis will tend to vary with starting phage titers and peak culture turbidities will also tend to vary with starting phage titers [52]. The resulting correlations tend to be with the logarithm of the starting phage titer [19,49,52]. The workflow is thus: add phages of unknown titer to a culture of known bacterial concentration → incubate with kinetic monitoring of culture optical density → determine the timing or other metric of the phage impact on the bacterial culture → compare that metric to a previously obtained calibration curve for that specific phage-host combination.

My overall impression of this approach is both favorable and pessimistic. On the one hand, estimation of starting phage titers seems to be readily accomplished. However, that assessment comes with five caveats:

- **Calibration requirements:** The assay requires prior generation of calibration curves for every phage genotype to be assayed, representing a substantial upfront investment.
- **Lysis inhibition complications:** Plaque-based assays are less impacted by the complication of lysis inhibition or any other complications to the shape of lysis profile curves (such complications can be seen in Figure 2 and Figure 3, respectively).
- **Precision limitations:** Plaque assays can more consistently provide substantial titering precision, e.g., as little as the square root of a plaque count [29] vs. sometimes two-fold differences for KOTE assays [52].
- **Time savings uncertain:** Though a primary utility of the KOTE approach is saving time in phage titer determinations, that time advantage can be lost given optimization of plaque-based titer determinations [132].
- **Greater equipment requirements:** Though KOTE assays can be less materials intensive, they are more equipment intensive, requiring access if they are going to be conveniently done to what generally are somewhat expensive incubating and shaking kinetic microtiter plate readers.

Notwithstanding these concerns, KOTE assays could be useful to the extent that future phage titer determinations become fully automated—perhaps in combination with localized, fully automated phage-production platforms to support phage therapy use [133].

## 6. Lysis from Without and Resistance to Lysis from Without

Lysis from without is a mechanism of premature bacterial lysis that is induced by high-multiplicity phage adsorption, e.g., 100 phages per bacterium [79,134]. This phenomenon appears to be caused, at least in certain phages, by over-exposure of bacterial cell envelopes to virion-associated peptidoglycan-degrading enzymes [25], that is, by the action of so-called ectolysins [135]. The result is an occurrence of bacterial lysis that is both premature relative to the normal timing of phage-induced lysis—described instead as a lysis from within [79]—and in which the normal phage infection processes are also truncated. The net result is a bacterial lysis that can be observed in bulk as a substantial, quite early reduction in the turbidity of the affected culture.

A recommended workflow for detecting lysis from without consists of: modifying bacterial physiology to prevent effective expression of resistance to lysis from without (e.g., starvation or use of metabolic inhibitors) → using sufficiently high numbers of bacteria that reductions in turbidity are easily observed → addition of a very high multiplicity of virions, such as 100 → kinetic monitoring of culture turbidity. The expectation is that lysis will be observed sooner than expected if it were based solely on lysis from within, where lysis from within should either be not possible or greatly delayed due to the absence or near-absence of bacterial metabolism.

### 6.1. Optical Observation of Lysis from Without

Visualization of premature phage-induced bacterial lysis, no matter how that lysis is monitored—such as in terms of optical density [136] or instead, e.g., microscopically [79]—should be viewed as a requirement when invoking claims of lysis from without. Indeed, bacterial death alone should not be thought of as evidence of lysis from without, no matter how high the multiplicity of phage adsorption, since bacterial death resulting from phage exposure can be expected regardless of the multiplicity of adsorption. One must also be careful to not confuse phage infection death following high multiplicity phage adsorption with actual phage adsorption-induced premature bacterial lysis, as these are not necessarily identical processes [137]. Consequently, cell death alone upon adsorption by high multiplicities of wild-type phages should not be viewed as evidence of lysis from without, even if accompanied by a lack of virion production.

**Figure 4:**
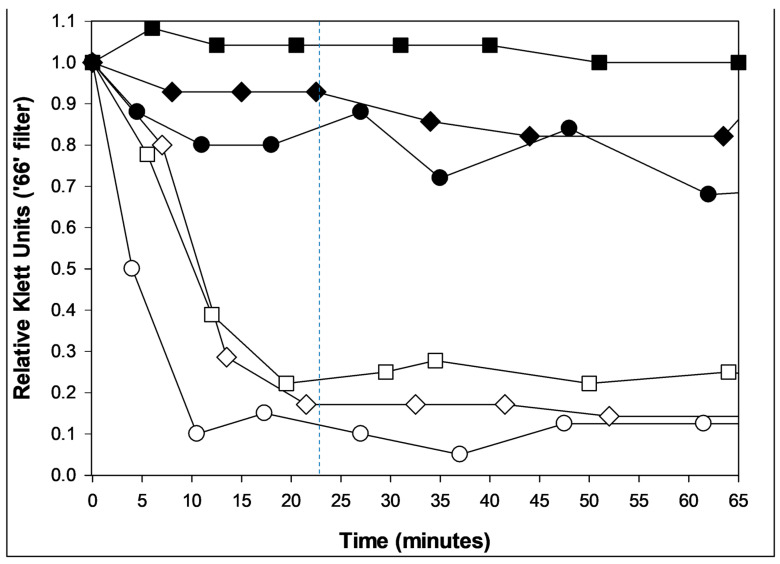
Lysis from without and resistance to lysis from without. Shown are coliphages T2 (circles, ○), T4 (squares, ☐), and T6 (diamonds, ◇). Primary phage adsorption (solid symbols; multiplicity of 3) was to metabolizing bacteria (*E. coli* K12) for five minutes to allow for early gene expression. These were then washed twice and suspended in M9 salts (a phosphate buffer [147]). Alternatively, no primary phages were supplied (open symbols). In either case, cell washing and resuspension in buffer was followed at time zero with the addition of a multiplicity of 100 of the same phage type. Solid symbols thus indicate a resistance to lysis from without [86,138] while open symbols indicate the occurrence of lysis from without. The latter lysis happens in roughly half as much time as expected with lysis from within (23.5 min for the latter [84], as approximated by the dashed, vertical line). Bacteria also should be only minimally metabolizing due to negligible carbon source availability; these phage infections therefore should be unable to produce substantial quantities of phage lysis-from-within proteins. The absence of lysis in the closed-symbol curves is presumably due to the presence of minimal carbon and energy to these phage infections though in addition all three of these phages are able to display lysis inhibition (Section 4.3; [93,95,148]). A single biological repeat is presented.

To claim lysis from without, observation of premature bacterial death instead must be accompanied by evidence of premature, phage-induced, substantial early drops in bacterial culture turbidity and/or microscopic observation of early cell lysis. Notably, Henry et al. [27] reported an absence of evidence for lysis from without with *Bacillus anthracis* phage Giraffe. See Figure 4, here, for optical density-based visualization of lysis from without. Also shown in that figure is phage-encoded *resistance* to lysis from without [138], the latter being a possibly lysis-inhibition-related mechanism that also requires phage gene expression to implement [86,95,97].

### 6.2. Additional Lysis-from-Without Caution

Lysis from without also is not readily demonstrable using phage application to, especially, immature bacterial lawns, e.g., [36], i.e., as in the course of higher-multiplicity spot testing [139,140]; this is versus spotting with lower multiplicities for phage titering purposes [37,132]. This is because even though high multiplicities of phages may be applied to plates as drops, as noted generally only one phage per bacterium is needed to kill and lyse a bacterium. Such spotting thereby generates a “zone of inhibition” of sorts under that spot, that is, what is traditionally described as a confluent lysis, and this occurs whether or not lysis from without has occurred. What occurs instead is the formation of a zone of inhibition generated by applying liquid rather than disk-associated addition of an antibacterial agent, e.g., [141]. See, however, Skusa et al. [142] for use of both “lysis zone” as a synonym and a disk-based phage-susceptibility assay. Note also the potential for phage-encoded endolysin enzymes to generate lysis zones without whole-phage involvement, though this effect tends to be limited to Gram-positive hosts [143,144,145,146].

Neither bacterial death nor lawn clearing alone therefore are reasonable indicators of lysis from without, even with the addition of high titers and multiplicities of phages. The converse *is* true, however: lysis from without represents a robust means of effecting bacterial death, though it results in a bacterial lysis that is not associated with production of new phages.

### 6.3. Non-Optical Assay for Resistance to Lysis from Without

Notwithstanding these various caveats, one *can* study lysis from without in terms of deaths, but of phage-infected rather than phage-uninfected bacteria. This involves infecting bacteria with phages—which should yield a specific number of plaque-forming units (PFUs) per this primary infection—and then following that up with a high multiplicity of phage adsorption (secondary adsorption). That, upon lysis from without, should result in substantial reductions in numbers of plaque-forming, phage-infected bacteria—so long as this lysis is achieved prior to the end of the phage eclipse period [31,149,150,151].

It is crucial in setting up these experiments that phage-infected bacteria can be distinguished, upon plating, from free virions. This can be done by centrifuging and then removing free phage-containing supernatant or instead employing adsorption- but not replication-competent secondary phages (particularly mutant phages rather than, e.g., UV-treated phages, the former so as to avoid multiplicity reactivation [152,153]). This approach of applying large numbers of virions to already phage-infected bacteria was developed toward characterizing what is known as phage-encoded resistance to lysis from without, which, like an ability to display lysis inhibition, can develop early in phage infections [138] (Figure 4). That is, given phage-expressed resistance to lysis from without, then reductions in plaque-forming units should not occur to as great an extent in the absence of such resistance. Especially phage T4 *spackle* mutants are defective in their display of resistance to lysis from without [25,136].

## 7. Phage Host-Range Determination

Lysis is the end stage of lytic infections, and substantial turbidity reduction of a culture is dependent on the lytic infection of a substantial fraction of the bacteria present. If phages are added to bacterial cultures with a multiplicity of less than one, then culture-wide bacterial lysis will occur only if the bacteria present also are susceptible to productive lytic infections by that phage type [55]. That is, only if the bacterial strain being tested is found within a phage’s host range—or, more specifically, a phage’s productive host range [154,155]—will substantial reductions in turbidity occur, if starting with a lower phage multiplicity. This section focusses on a number of issues related to optical density-based assessments of phage host range, with host range defined especially in terms of a phage’s ability to both productively and lytically infect specific bacterial strains.

The general workflow should consist of: Phage addition at a multiplicity of somewhat less than one (e.g., 0.1 or lower) → incubation with kinetic monitoring of culture optical density → observation of phage impacts on culture turbidity that are consistent with expected phage latent periods. For example, if one round of phage infection and lysis is expected before phage impact on culture turbidity is noticeable—such as starting with a phage multiplicity of 0.01 and an expected phage burst size of 100—then the timing of that impact should be roughly equal to no less than one latent period length and ideally to no more than two latent periods following phage addition. As will be considered below, substantial delays beyond the latter can be indicative of a phage host-range false-positive result.

### 7.1. Optical Density-Based Phage Host-Range Studies

Xie et al. [55] describe a strategy for using microtiter plates, and determinations of changes in the optical density of bacterial cultures over time, to infer that a bacterium is found within a phage’s host range. This involved the addition of phages at four different concentrations—10^8^, 10^7^, and 10^6^, plus 0 PFUs/mL (Plaque-Forming Units/mL)—to two different concentrations of bacteria (10^8^ and 10^5^ CFUs/mL, for Colony-Forming Units), which ideally will be in mid-log phase at the start of assays (Section 2.5). As noted, with lower starting ratios of phages to bacteria, culture-wide bacterial lysis should occur only if the bacterium is found within the phage’s productive host range. Ratios of phages to bacteria that are too small, however, may result instead in a lack of culture-wide bacterial lysis, i.e., as discussed in Section 2.2.

Consequently, there can exist at least four possible outcomes to phage host-range determinations employing kinetic optical density measures, particularly if we ignore for now the potential for phages to mutate to host-range variants (Section 7.2 and Section 8). These are:

- Productive infection host-range positives (culture clearing starting with a lower phage-to-bacterium ratio),
- Culture clearing but without evidence from optical density data of productive infection (i.e., starting with a phage-to-bacterium ratio of greater than 1),
- False productive-host-range negatives stemming from too low starting phage-to-bacterium ratios (Section 2.2), and
- True host-range negatives as evidenced by a consistent lack of culture-wide bacterial lysis, particularly as based on experiments initiated with a variety of phage multiplicities.

Overall, this means that there could be differences in the perception of a phage’s optical density-based host range—as stemming from whether or not culture-wide bacterial lysis occurs—that are dependent on the magnitude of the starting ratio of phages to bacteria, i.e., with MOIinput.

Alternatively, as considered in the following section, there can also be

- Productive infection host-range false positives due to the replication of phage host-range mutants and
- Productive infection host-range “false” false (and thus, ‘pseudofalse’) positives due to slow culture lysis by wild-type phages (host-range positives mimicking host-range negatives).

The latter refers to a result that mimics a false positive. This is as appears to be due to the actions of a phage host-range mutant, but is actually a true host-range positive—it is just a wild-type phage that displays poor growth characteristics on a given test bacterium (Section 7.2.3).

The next section (Section 7.2) elaborates on how either phage host-range mutants (resulting in false host-range positives) or poor phage population growth by wild-type phages (resulting in pseudofalse host-range positives) can result in delayed phage population growth. This is relevant as it can make the assignment of a test bacterium to a phage’s host range ambiguous. See Table 2 for a summary of possible outcomes from optical density-based phage host-range determinations and their interpretations.

### 7.2. Beware Phage Host-Range Mutants—False-Positive Outcomes

Optical density-based host-range determination requires distinguishing true host-range positives from false host-range positives. With a true host-range positive, the wild-type phage is able to productively infect the test bacterium, whereas false positives can arise when rare phage host-range mutants rather than wild-type phages are responsible for the observed bacterial lysis.

#### 7.2.1. Distinguishing True from False Positives Using Timings

The key feature distinguishing true from false host-range positives is lysis timing. With true positives, the phage impact on a tested bacterial culture should occur somewhat sooner than for false positives. “Somewhat sooner” can be determined by comparing the phage’s lysis kinetics on a new bacterial host with those seen when infecting an established host, one that the phage is known to infect well. When in doubt, however, consider using an alternative approach to testing phage host range, such as plaquing ability (Section 7.3 and Section 7.4).

The challenge is that even wild-type phages can, under various circumstances, display a delayed lysis. This is despite a host bacterium technically falling within its host range, resulting in the noted pseudofalse positives (which could also be described as displaying a lower antibacterial virulence against a given host [22,55]). Therefore, two distinct mechanisms exist that can produce delayed lysis timing, requiring different interpretations: true false positives due to phage host-range mutants (Section 7.2.2) and pseudofalse positives that are not due to phage host-range mutants (Section 7.2.3). The difficulty when faced with seemingly false-positive results is then distinguishing between these two possibilities.

**Table 2:** Phage biological determinations based on optical density.

Starting MOI ^1^	Timing	Explanation	HR ^2^ Interpretation
Low	No lysis and no deviation ^3^	Lack of productive infection or insufficient phage antibacterial virulence	Either host range negative (no phage productivity; Section 2.2) or false negative (low phage virulence; Section 7.2.3)
Low	No lysis but still relatively early deviation ^3^	Productive infection but with slow or no lysis especially at higher bacterial densities	True host-range positive (but see Section 2.2); could be lysis inhibition (could be designated as intermediate bacterial sensitivity; Section 7.2.4)
Low to moderate	Tens of minutes to a few hours until lysis	Productive infection	True host-range positive; see, however, “Moderate to high” MOI, below
Moderate	Few to many hours until lysis but relatively early deviation ^3^	Lysis inhibition ^4^	True host-range positive ^5^ but could be mistaken for intermediate bacterial sensitivity (Section 7.2.4)
Moderate to high	Few to many hours until lysis but *without* relatively early deviation ^3^	Host-range mutants	Host-range false positive (Section 7.2.2)
High	Normal latent period	Bacteriolytic infection	True host-range positive ^6^
High	Few to many hours until lysis but with early deviation ^3^	Lysis inhibition ^4^	True host-range positive ^6^ but could be mistaken for intermediate bacterial sensitivity (Section 7.2.4)
Very high	Very early lysis	Lysis from without	Ambiguous; possible host-range false positive but probably host-range true positive (Section 6)

^1^ MOI stands for Multiplicity of infection, defined here as the ratio of added phages to added bacteria (added PFUs/added CFUs), also described as MOI_input_ [89]. A low MOI is <<1, a moderate MOI is <1, a high MOI is >>1 and very high is >>>1, e.g., 100. An MOI of 1 is somewhere between moderate and high. ^2^ Host range (HR). ^3^ Deviation of with-phage optical density from without-phage optical density. ^4^ Especially at higher culture turbidities, corresponding, e.g., to somewhat greater than 10^8^ bacteria/mL, some phages can also display delays in lysis that are not necessarily associated with lysis inhibition sensu stricto [21,49,52,74,112,113,114,115,116,156]. ^5^ Could also be slow or no lysis, especially at higher starting cell densities. ^6^ Could also be (and probably is) a productive infection; this is not definitive proof that new phage virions are released upon lysis, though that can be tested via post-lysis plaquing.

An alternative metric to culture-wide bacterial lysis—as a means of determining the timing of a phage’s impact on the bacterial culture—is the point at which the phage-containing culture’s turbidity deviates from that of the phage-free control [49,113,114,157]. The advantage of this metric is that it indicates a phage impact on a bacterial culture even if culture-wide bacterial lysis itself is substantially delayed. For example, the T4D curve in Figure 2 (☐) shows such deviation around the 1 h mark, indicating productive infection, even though culture-wide bacterial lysis is delayed until about 4 h. This deviation is seen—and seen at that point in time—because the bacterium used is found within T4D’s host range (*E. coli* B). See also Figure 3 as well as Section 7.2.4. I am currently working on an objective approach to determining these points of deviation, even when working with noisy optical-density data.

Thus—whether in terms of the timing of culture-wide bacterial lysis or instead the timing of deviation—the challenge in distinguishing true- from false-positive host-range results is one of recognizing that not all observed phage impacts are necessarily due to the action of the wild-type phage being tested (Section 7.2.2). Nonetheless, it is also possible for wild-type phages under certain circumstances to mimic false-positive delays (Section 7.2.3). Suggestions for mitigating these issues are provided in Section 7.2.5.

#### 7.2.2. Delays Associated with Phage Host-Range Mutants

Phage host-range mutants can be inherently present at the start of most experiments, and they usually are present if stocks contain sufficient numbers of virions. However, without selection, phage host-range mutants are expected to be present at much lower titers than their wild-type phage parents [121,158,159,160,161,162,163]. Given those lower starting numbers, if those are the only phages able to bring about a culture-wide bacterial lysis (rather than the wild-type parent), then that lysis should occur somewhat later than expected. For example, if one starts with a culture with 10^1^ phages rather than, e.g., 10^6^, then multiple additional rounds of phage infection and lysis will be required before phage numbers catch up with those of bacteria. Assuming in this example that the phage latent period is 20 min, and that three additional rounds of infection are required, that would represent a one-hour delay for the phage host-range mutant relative to wild type.

If the fraction of host-range mutants is even smaller, or the mutant is less effective in phage population growth—relative to what would be expected from its parent on its original host—then the delay can be longer. The longer the delay, of course, the more readily the delay should be recognizable as distinct from the impact that wild-type phages have when infecting a bacterium that is found within their host range. This suggests a utility of first using optical density as a means of distinguishing likely true host-range positives (minimal delays) from possible host-range false positives (more substantial delays but still an impact), but then testing the latter by alternative means (Section 7.3 and Section 7.4).

This process—of host resistance followed by selection for a phage host-range mutant—mimics phage-bacterial antagonistic coevolution [164,165]. This is evolution of phage resistance by bacteria that is countered by phage evolution that overcomes that resistance, and so on. Here, though, such false host-range positives would be starting with a bacterium that is already phage-resistant, but with this followed by a rise in the frequency of phage host-range mutants. This selection for a host-range mutant over wild-type is expected to take time, however, which would be the origin of more-substantial delays in phage impact on a bacterial culture—phage population growth is not an instantaneous process.

#### 7.2.3. Other Causes of Delayed or Altered Lysis Timing

Phage host-range mutants unfortunately are not the only possible source of delays. For instance:

- Reductions in wild-type starting phage titers will result in delays in culture-wide bacterial lysis (Section 5), or in failures of cultures to lyse at all (Section 2.2).
- Slowing phage population growth will also result in delays, what can be dubbed instead as a reduced antibacterial virulence [22,55] or reduced infection vigor [154].
- Lysis inhibition will of course result in delays in overall lysis (Section 4), but not necessarily also delays in deviation (Figure 2; Section 7.2.4).
- Bacteria that have grown to higher concentrations can also slow rates of phage-induced bacterial lysis [21,22,49,52,55,74,76,112,113,114,115,116,117].

Collectively, these different types of delays could be misinterpreted as false positives—as due to the action of phage host-range mutants—when they actually are true positives, just with delayed lysis kinetics (i.e., pseudofalse positives/lower-virulence phages on the specific bacterial host). This ambiguity again points to the utility of using alternative means to test host-range results if those results are not obviously positive nor obviously negative.

Too-early lysis, in contrast, could be due to lysis from without (Section 6), representing a different type of false-positive result. Specifically, lysis from without can serve only as an indication of a bacterium being within a phage’s adsorptive host range. Lysis due solely to lysis from without, however, can be avoided by starting with low phage multiplicities, which are preferable regardless for phage host-range testing by optical density means (Section 7.2.5).

#### 7.2.4. Bacterial Sensitivity

Xie et al. [55], in their optical density-based host-range determinations, use a concept of bacterial sensitivity to distinguish (i) within host range from (ii) not within host range from (iii) in-between or intermediate. From this perspective, slower phage population growth, resulting in slower lysis, may give rise to an intermediate bacterial sensitivity status. See also Storms et al. [22] for equivalent underlying calculations to those of Xie et al. That is, the Xie et al. “liquid assay score” appears to be equivalent to the Storms et al. “local virulence”. Phages that display lysis inhibition in particular might be labeled as giving rise to only intermediate bacterial sensitivity. For that, see again Storms et al. [22]. There, a designation of lower, but not lowest virulence for phage T4, appears to be equivalent to what would presumably be described as an intermediate bacterial sensitivity by Xie et al.

This designation of intermediate bacterial sensitivity or lower virulence, though, could be false, meaning that bacterial sensitivity in fact can be higher than appreciated by the Xie et al. [55] and Storms et al. [22] measures. This is a result of lysis inhibition being associated with very long latent periods as well as potentially slower lysis once initiated (Section 4). That greater-than-intermediate bacterial sensitivity is evidenced by the point of deviation of lysis-inhibited cultures from cultures that are not phage-infected. See Figure 2 for an example of this phenomenon, where deviation of the lysis-inhibited curve—the point at which most bacteria likely are now phage-infected—occurs around one hour, whereas culture-wide bacterial lysis does not occur until after four hours. Specifically, in that same figure, the phage T4 *r* mutant is clearly not giving rise to an intermediate bacterial sensitivity, but nevertheless displays a deviation starting at essentially the same time as the much slower-lysing wild-type T4.

#### 7.2.5. Recommendations

Taken together, these various issues regarding potential host-range false positive results point to a utility in performing turbidity-based host-range determinations that adhere to the following suggestions:

- Using kinetic rather than endpoint assays;
- Employing a variety of phage multiplicities [55,166] to better avoid ambiguous results, though approximately 0.01 phages-to-bacteria in many cases might be ideal (see the following paragraphs);
- Not starting with excessive phage multiplicities, in part because this introduces potential host-range mutant phages in higher numbers (below), but also because if wild-type phages are sufficiently high in starting titer, then lysis but without virion production could give a false-positive result (Section 6);
- Not starting with excessive bacterial concentrations so that cultures do not enter stationary phase before phage population growth has caught up with bacterial growth;
- Looking for relatively early deviation of phage-containing curves from those of bacteria, especially when starting with lower phage numbers, rather than explicitly requiring relatively early culture-wide bacterial lysis;
- Truncating experiments such that any impacts of delayed phage population growth never manifest, though this would also constitute a more stringent definition of phage host range, which should be recognized as such (i.e., see Section 7.2.3); and
- Testing ambiguous results by alternative means.

An additional issue stems from the volume of test cultures, since the number of starting phages will equal volume × phage multiplicity × bacterial concentration. Ideally, the number of virions introduced into a culture will be sufficiently low that it is unlikely that phage host-range mutants will be present at the start of experiments. If those phage mutants are not initially present, and phages otherwise are unable to replicate (because the test bacteria are found outside of their host range), then mutant phages in most cases should never arise.

If one started with 10^7^ bacteria/mL and applied phages at a multiplicity of 0.01, then that would be 10^5^ phages/mL. If the culture consists of only 100 μL, then that results in only 10^4^ phages added in total. That in most cases—RNA phages potentially being an exception [167]—should be sufficient to prevent an initial presence of phage host-range mutants. Smaller volumes therefore should be viewed as preferable for optical density-based host-range determinations, along with starting with lower (though not excessively low; Section 2.2) phage multiplicities.

### 7.3. Host-Range Mutants Not Just an Issue with Optical Density-Based Methods

This potential to conflate the impact of wild-type phages with those of rare host-range mutants can be an issue with other approaches to host-range testing. For example, phage efficiencies of plating (EOPs) [139,140] can provide results that do not always coincide with optical density-based methods [168]. Consistently, it is important to distinguish high EOPs—which indicate that a bacterium is found within a phage’s host range—from low EOPs, the latter as potentially implying that a bacterium is not found within the host range of the phage being tested. Specifically, low EOPs can be associated with plaquing by a phage’s host-range mutants. That essentially is the same phenomenon as considered in the previous section (Section 7.2), though with phage host-range mutants being countable here as PFUs [169], i.e., as associated with low EOPs, rather than being indicated as a later than expected onset of culture-wide bacterial lysis. Alternatively, EOP reductions can be due to bacterial restriction-modification systems, but can be overcome at low rates through phage epigenetic changes [29], or EOP reductions due simply the above-noted reduced infection vigor [154].

For cases of low EOP due to reduced infection vigor, an alternative plaquing approach to EOP determination exists, which can be less reliant on infection vigor. This is called an Efficiency of Center of Infection or ECOI assay [170,171,172]. It involves pre-adsorbing phages to a test host, removing unadsorbed virions, and plating using indicator bacteria on which the phage is known to readily plaque. With this approach, there is no need for a phage to effectively form a plaque on a lawn of the test bacteria. Instead, the phage needs only to successfully infect and lyse the test bacterium to result in plaque formation.

A similar concern may be seen with the use of spot testing to determine phage host ranges [140] (Section 6.2). The likelihood of clearing (confluent lysis) being due to phage host-range mutants, however, should increase if more phages are applied as well as if large plaques are produced by individual phage host-range mutants. Indeed, Welkos et al. [173] specifically reported an increase in spot testing “sensitivity” under conditions where more phages were applied, along with when phages have the potential to grow larger plaques. For the latter, they explicitly indicated that “lysis [meaning spot formation] was more complete due to formation of larger plaques”. This again is highly suggestive that host-range mutants could be responsible for their, I speculate, *false* host-range positive results, rather than the majority-applied wild-type phages being responsible for spot clearing.

Especially if it is possible for relatively few plaques to generate a clear spot, then the more total phages that have been applied, the greater the potential for spotting host-range false positives that are due to rare phage host range-mutants falsely indicating spot clearing despite being present in relatively low numbers. Similarly, if they are sufficiently abundant at the start of experiments, then phage host-range mutants also may be able to cause relatively rapid reductions in the turbidity of liquid cultures (Section 7.2.2).

### 7.4. Testing for Host-Range Mutants

Ultimately, it should be possible to isolate phages from the late-lysing (or low-EOP) cultures and then repeat optical density-based experiments using those phages. In that case, if late lysis or deviation from no-phage bacterial growth curves had been attributable to phage host-range mutants, then (at least ideally) those newly isolated phages should no longer display that late lysis or turbidity deviation when applied at higher initial titers. They should also no longer display EOPs that are as low as before. That is, a host-range mutant able to infect a new host, relative to the phage’s parental genotype, should now display more effective within-host-range properties than the parental wild type on the same host.

Alternatively, Niu et al. [54,61,62] used Appelmans’ titering method (Section 2.4) to assess phage host range characteristics. Specifically, a phage infecting a bacterium within its host range should be able to lyse a culture of those bacteria at a higher phage dilution than a bacterium that is not within a phage’s host range. This approach appears to be somewhat equivalent to higher EOPs resulting when a bacterium is closer to within a phage’s host range, substituting numbers of plaques at a given dilution with numbers of cleared tubes.

## 8. Bacterial Evolution of Phage Resistance

**Figure 5:**
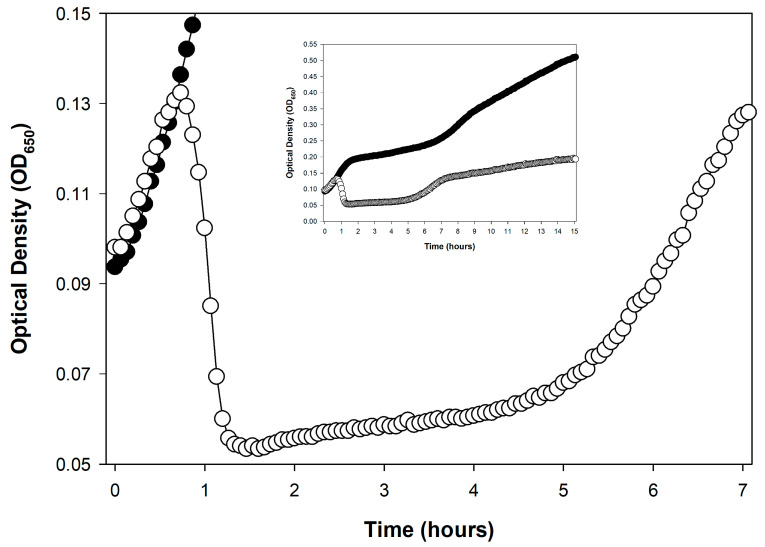
Grow back of a bacterial culture following phage-induced, culture-wide bacterial lysis. This growth presumably is associated with the evolution of phage-resistant bacterial mutants, as the phage used, coliphage T3, is not temperate. The experiment is otherwise equivalent to that shown in Figure 1, except that it is shown here over a longer span of time. With phage T3 present is indicated as open circles, ○, and *E. coli* B growing alone is shown as closed circles, ●. See Appendix A for additional methods.

For many combinations of phages and bacteria, cultures can contain phage-resistant bacterial mutants [121,158,159,160,161,162,163]. These mutants can be detected as post-lysis rises in culture turbidity; alternatively, so can phage initiation of lysogenic cycles result in regrowth of phage-resistant bacteria [52]. This bacterial grow back typically follows, by many hours, initial phage-induced reductions in culture optical density [55,174,175,176,177,178,179] (Figure 5). Though not explicitly phage-induced bacterial lysis, the grow back of cultures can be studied using equivalent optical density-based methods, and can also be relevant to interpreting those experiments, hence inclusion of the topic here. The workflow consists of simply applying phages to bacteria at population sizes where resistance mutations are likely to occur, and then monitoring cultures for extended periods, recognizing that stopping observations too early can result in false negatives for the presence of phage-resistant bacteria.

### 8.1. Kinetics of Bacterial Grow Back

The timing of bacterial grow back will be dependent not just on the likelihood of bacterial mutation to phage resistance but also on the doubling times of resulting bacterial mutants. Therefore, if both mutation frequencies and mutant growth rates are high, then we expect that a greater number of resistant bacteria will be present at the point where their phage-sensitive competitors have been wiped out. After that, regrowth will be noticeable sooner. Alternatively, if growth rates of phage-resistant bacterial mutants are slow relative to their wild-type parent, then even for the same mutation rates, mutant frequencies will be lower at the point of culture-wide lysis of phage-sensitive bacteria. Consequently, it should take longer overall until regrowth is observed turbidimetrically for those slower-growing mutants.

Mutant numbers, that is, will tend to start at one cell per culture and then increase in number over time via binary fission. The number of mutants present at any given time therefore will be a function of both when that first mutant arrived and then how fast that mutant subsequently divided. Fortunately, it is possible to distinguish slower-growing resistant bacterial mutants from faster-growing resistant mutants based on the slope of the recovering optical densities, i.e., as graphed against time. If doubling times are very long, however, then these slow-growing mutants may not even become detectable until after relatively long incubations. The turbidity that host-range mutants are able to attain is also relevant to their characterization, with peak turbidity in many cases somewhat lower than that attained by parental bacteria that have not been exposed to phages.

### 8.2. Likelihood of Bacterial Mutation to Phage Resistance

Whether phage resistance is going to arise at all is dependent on the frequency with which bacteria are able to mutate to this resistance. That in turn means that resistance occurrence will be a function of whether phage-treated cultures contain sufficient numbers of phage-sensitive bacteria to mutate to resistance with sufficient probability [180,181,182]. Fortunately, 100 or 200 µL, the volumes typically found in the wells of 96-well plates, often (but not always) will contain sufficient numbers of bacteria to result in mutation to phage resistance with relatively high likelihood. This is assuming that the phage-sensitive bacteria have been allowed to grow to sufficiently high concentrations before succumbing en masse to phage attack.

**Figure 6:**
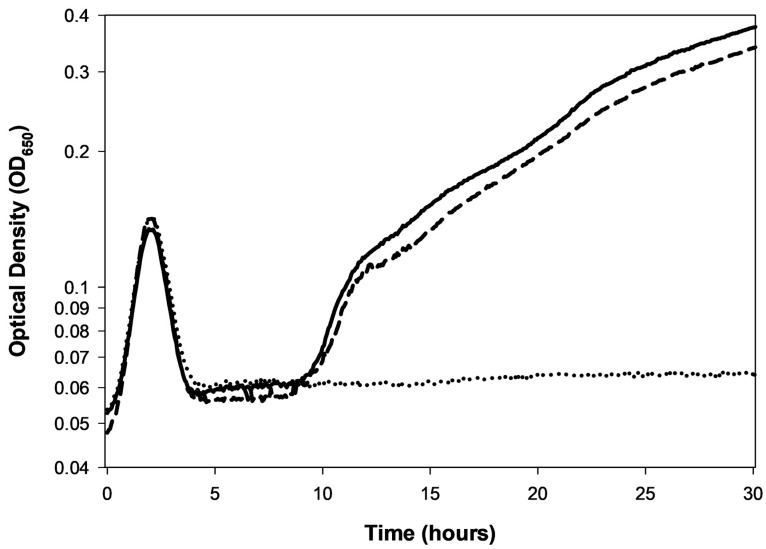
Stochastic occurrence of phage-resistant bacterial mutations and culture regrowth. Shown are three technical repeats initiated from the same phage T5-infected culture that was then split three ways (solid, dashed, and dotted). Phage resistance is seen as the rise in culture turbidity in two wells following phage-induced bacterial lysis (solid and dashed lines). This is seen starting around 10 h and is equivalent to the culture grow back observed in Figure 5 as well as in Figure 2. By contrast, note the lack of such grow back seen in the third well (dotted-lined curve). Differences like this between technical repeats based on the same split culture is unusual. It should occur only, for instance, if the phage-resistant mutant is rare at the point of splitting cultures. Alternatively, though even less likely, this can occur if separate but otherwise equivalent bacterial mutations were to arise at approximately the same time, but in only two of three separated cultures. Nonetheless, in this experiment, a resistant mutation never arose in one of the three wells (dotted curve). As the bacterial population was substantially diminished following phage infection, around 2–3 h into the experiment, this made subsequent mutation to phage resistance much less likely.

This dependence on sufficient numbers of bacteria means that a failure of bacteria to grow back following phage-mediated culture-wide bacterial lysis can have mutation-to-phage-resistance false-negative potential. This is a failure of a given bacterial strain to evolve resistance to a given phage isolate, and can be seen particularly if testing is done using smaller individual volumes (Figure 6). Note nonetheless that total volumes tested can be increased even when using 96-well microtiter plates simply by using more wells per phage-bacterium combination, e.g., 10 wells rather than 1. If the interest is in determining the likelihood of mutation to phage resistance, do not forget also to initiate bacterial cultures for experiment repetitions (biological repeats) from different overnights. This is because individual overnights initiated from separate bacterial colonies can vary in the number of phage-resistant bacterial mutants they carry [158].

Alternatively, note that slower-growing phage-resistant bacterial mutants may acquire compensatory mutations that can result in faster bacterial population growth [183]. Those mutations would be expected to arise particularly nearer to the point where this growth is observable via optical density measurements. This is because the more phage-resistant bacteria that are present in a culture, then not just the higher the culture’s turbidity, but also the greater the potential for any random mutation to occur.

An additional complication can occur to the extent that bacterial cultures have reached, prior to their lysis, sufficiently high densities that significant reductions in nutrients and/or oxygen have occurred. This is an issue that could be particularly problematic with phages that display long lysis-inhibited latent periods (Section 4), i.e., as higher levels of nutrient or oxygen use would continue in these cultures for many hours following the initiation of phage infection of a majority of bacteria. Even if phage-resistant bacteria are present, their population growth to detectable numbers thus may be slowed due to the extended presence of high concentrations of these still-metabolizing, lytically phage-infected bacteria. Although not definitive, nonetheless again see Figure 2, for example, where bacterial growth is observed with the *r* mutant a few hours prior to when it is seen with the lysis inhibition-displaying wild-type phage T4.

### 8.3. Host Range vs. Resistance Suppression

It is important to emphasize that delays in bacterial regrowth following phage-induced lysis are measures of resistance suppression—defined here as a slowing or prevention of the growth of phage-resistant bacteria—and do not serve as an indicator of a phage’s host range. This section further explores this distinction between phage host range and phage ability to suppress bacterial evolution of phage resistance.

#### 8.3.1. The PHIDA Tool (“Phage-Host Interaction Data Analyzer”)

The following variables—culture volume, likelihood of mutation, timing of mutation, peak numbers reached by phage-sensitive bacteria, and rates of mutant bacterial growth—are all relevant to the PHIDA tool presented by Martinez-Soto et al. [184]. PHIDA stands for “Phage–Host Interaction Data Analyzer”. It measures timings of delay in bacterial growth (to OD_600_ = 0.2) relative to a phage-less control. Alternatively, maximum optical density (OD_max_) is compared if cultures fail to lyse completely over the course of experiments.

A phage MOI of 1 is used and three outcomes are distinguished:

- “A”, culture-wide bacterial lysis and no culture regrowth (effectively infinite delay).
- “B”, culture-wide bacterial lysis followed by culture regrowth (delay is quantified).
- “C”, no or incomplete culture-wide bacterial lysis (ODmax is determined).

The primary difference between the “A” and “B” scenarios, on the one hand, and the “C” scenario on the other, is phage antibacterial virulence [2,22,49,53,54,55,56,57]. Specifically, “C” is either displaying low virulence (resulting in poor culture lysis) or instead the bacterium being tested is found outside of a phage’s host range (no lysis and no deviation). See though the concern over phage host-range mutations discussed here in Section 7.

Of most interest to this section—exploring bacterial mutation to phage resistance—is the Martinez-Soto et al. [184] “B” result. It is there that delays are seen between the phage-treated and bacteria-only control in terms of reaching the threshold OD. These delays should vary in length as a function of the variables listed at the top of this section, especially differences in likelihoods of mutation to phage resistance, differences in phage antibacterial virulence (since that will have some impact on peak densities of sensitive bacteria), and rates of growth of phage-resistant bacteria. What the PHIDA tool does is to automate calculations of the lengths of these delays.

The PHIDA tool nonetheless is presented as a means of determining what the authors describe as a “Host-Range index”, or *HR*i. However, in terms of either phage host range or antibacterial virulence, there could exist no detectable differences between the “A” result (no regrowth) and the “B” result (delayed regrowth). That is, in principle two phages can possess the same antibacterial virulence against a given bacterial strain that is found within both of their host ranges, but still display differences in their ability to suppress bacterial evolution of resistance. The PHIDA tool, by measuring delays in resistance observation, therefore, does not evaluate phage host range.

#### 8.3.2. A Resistance-Suppression Index

What is being measured for “A” and “B” is therefore more strictly a “Resistance-Suppression index”, or “*RS_i_*”. The concept of *RS_i_* should be applicable not just to individual phages but also to phage cocktails or to phages in combination with other antibacterials [182]. Kim et al. [185] describe a similar “Suppression index” that instead employs area under the curve (AUC) calculations rather than just the timing of regrowth, but which they use to assess the impact of phage cocktails. Though similar as a measure of resistance occurrence, the AUC calculation takes more into account than just the timing of regrowth. This includes the extent of turbidity recovered as well as, by necessity, the duration of the experiment (as AUC calculations always have an interval within which they are calculated).

Li et al. [186] provide an important illustration of this suppression index in a study predating that of Kim et al. [185]. There, the extent of growth can be seen to vary depending on how many and what phages the targeted bacterium is exposed to. In all cases, however, turbidity never recovered to that seen with the uninfected bacterial control, suggesting that differences in resistant-mutant fitness can be provisionally assessed using this approach. The timing of recovery also does not seem to correlate with resulting peak culture turbidities (as I inferred by visual inspection), suggesting also limitations to relying on just the timing of culture growth to assess resistance suppression.

Incubation times required to obtain AUC-type results can be longer than those needed to determine *RS_i_*, though longer incubation times nonetheless can be useful for both. Li et al. [186], for example, did not observe substantial regrowth until after 15–30 h working with *Vibrio alginolyticus*. AUC-based methods therefore can require somewhat longer incubations than *RS_i_* determinations, e.g., twice as long, but extended incubations can also supply more information as to the characteristics of the resulting phage-resistant bacterial mutants, including especially peak regrowth turbidities.

#### 8.3.3. Bacterial Hold Time

That same quantity—determining phage-resistance suppression—has also been determined by Parmar et al. [187], in what they describe as an optical density-based “Hold time”. This is “the time through which bacterial growth is inhibited by phage as compared to controls.” This approach is derived from their previous use of a Biolog Omnilog™ system [188]. Thus, an alternative, metabolism-based approach—using tetrazolium salt—seems to have been developed subsequent to the work of Martinez-Soto et al. [184] (Section 8.3.1), with the metabolism-based approach compared with using optical density in the later Parmar et al. study [187]. In both cases, they describe this approach as a means toward “phage susceptibility testing”, which can be interpreted as determining a phage’s host range. As emphasized above, however (Section 8.3.2), delays in the regrowth of culture turbidities represent the time until phage resistance evolution becomes apparent, rather than representing a host-range determination.

Appelmans’ protocol (Section 2.4) could serve a similar role. Specifically, though still confusing the concepts of host range, virulence, and phage-resistance suppression, Daubie et al. [34] suggest—referring to Appelmans’—that, “After overnight incubation, the culture is watched for visible lysis, indicating productive infection. Then, over a three-day time span, the appearance of ‘re-growth’ by resistant mutants is repeatedly watched for. The longer the medium stays clear, the more active the phage is against that strain.” That, however, is explicitly a measure solely of the duration of a phage’s ability to suppress the occurrence of bacterial resistance to the treating phage.

As a potential complication, note that over multiple days, it is conceivable that the phage host-range mutants could give rise to a perceived further delay in culture regrowth (Section 7.3, re: antagonistic coevolution).

### 8.4. Problems of False Negatives and Mechanistic Uncertainty

Optical density-based approaches to detecting bacterial evolution of phage resistance can be prone to false-negative results. That is, they may show an absence of evolution of phage resistance in systems where evolution of phage resistance should be possible. There are at least three possible sources of that error:

- Cultures never reach sufficient bacterial numbers such that mutation to phage resistance is likely (e.g., Figure 6).
- Insufficiently long incubation times such that more slowly growing bacterial resistance mutants fail to be detected (e.g., Figure 2).
- Potential for antagonistic coevolution (Section 7.2), where within a single culture bacterial evolution of phage resistance could be countered by subsequent phage host-range evolution.

The potential for bacterial evolution of resistance to phages is certainly relevant. Optical density-based approaches to assessing this potential likely can provide greater throughput than plating-based analyses [121,158]. At the same time, however, results of these assays may have more complex underpinnings than may be fully appreciated, particularly regarding underlying reasons for differences in culture regrowth delays.

## 9. Conclusions

Lysis of liquid bacterial cultures is as basic to phage study as the production of phage plaques [172]. Relative to phage plaques, however, analysis of liquid cultures is more easily automated. This includes—though only partially without robotics—via the use of kinetic, incubating, and shaking microtiter plate readers. An alternative is the use of the Omnilog^TM^ system which can run and analyze up to 50 96-well plates simultaneously [27,177,189,190]. With these optical analyses we can (i) titer phage stocks (Section 2 and Section 5); (ii) determine how fast phages can accomplish culture-wide bacterial lysis, whether in single or multiple steps (Section 3); (iii) observe lysis inhibition (Section 4); (iv) detect lysis from without as well as resistance to lysis from without (Section 6); (v) explore whether a phage can successfully infect at all (host-range determination; Section 7); and (vi) assess the potential for bacteria to evolve phage resistance (Section 8).

These various determinations are nonetheless not always trivially interpreted. Reasons include as due to the noted resistance evolution (Section 8), and the noted lysis inhibition (Section 4). Also potentially problematic is phage host-range evolution, which, at least in principle, can result in the lysis of cultures that otherwise are not expected to lyse (Section 7). The presence of those phenomena, however, contributes to a greater power of kinetic analyses over endpoint determinations (Section 2.3). Notwithstanding these complications, phage characterization can be greatly aided by optical density-based analyses. This includes in terms of labor savings over one-step growth experiments or phage host-range determinations.

For researchers new to these approaches, the following workflow is recommended: (i) Begin with simple lysis timing determinations using kinetic analysis to establish baseline phage behavior; (ii) verify that the phage system does not display lysis inhibition or develop workarounds if it does; (iii) only then proceed to more complex applications such as host-range determination or resistance evolution studies. Throughout, kinetic rather than endpoint determinations should be strongly preferred, as they provide far richer information and help avoid misinterpretation due to bacterial resistance evolution or delayed lysis phenomena. Such optical density-based phage characterization in any case is becoming increasingly common in conjunction with the growing prevalence in laboratories of kinetic microtiter plate readers.

## Figures and Tables

**Table 1:** Terms associated with optical density-based approaches to phage characterization.

Phenomenon	Description
Wavelength of light	Function of energy of photons; denotes colors within the visible spectrum, e.g., 400 nm (violet; higher energy) to 700 nm (red; lower energy).
Light intensity	Number of photons received by a detector per unit time but varying as a function of wavelength.
Light intensity detectors	Instruments that detect light intensity at specific wavelengths such as colorimeters, spectrophotometers, nephelometers, and turbidimeters.
Turbidimetry	Determination of degrees of scattering of light as resulting in declines in light intensity.
Colorimetry	As used here, the determination of concentrations of substances based on their ability to reduce the intensity of light.
Optical density (OD)	Degree of interference with the passage of light as typically defined in terms of a specific wavelength; a medium has an optical density that, unless it is transparent, has a value that is greater than 0.
Endpoint assay	Single measurement following some previously specified duration of incubation.
Kinetic assay	Multiple measurements taken over time, such as of the optical density of a culture, as determined over the duration of an incubation.
Lysis	Destruction of a bacterial cell envelope such as due to the action of cell wall- and membrane-disrupting phage-encoded proteins.
Lysis profile	Graphical representation of the kinetic impact of a lytic infection on a bacterial culture over time in terms of that culture’s turbidity.
Lysis from within	Phage-induced bacterial lysis as mediated by intracellularly located phage proteins. This is the lysis normally observed with lysis profiles.
Lysis from without	Premature lysis seen with some phages resulting from rapid, high-multiplicity virion adsorption without virion production.
Lytic phage	Bacterial virus for which successful virion-productive infections end in lysis rather than being associated with continuous virion release.
Culture-wide bacterial lysis	Conversion of a turbid bacterial culture to or nearer to the optical density of uninoculated broth.
Lytic cycle	Productive phage infection that ends with virion release and which follows either virion adsorption or prophage induction.
Lysogenic cycle	Phage infection by a temperate phage that is not virion productive but in which the phage genome replicates as a prophage.
Induced lytic cycle	Phage lytic cycle associated with conversion of a lysogenic cycle to a virion-productive phage infection that ends in lysis.
Purely lytic cycle	Lytic cycle that begins with phage adsorption rather than with prophage induction; this contrasts with an “induced lytic cycle”.
Latent period	Duration of a lytic cycle, e.g., as determined by employing either lysis profile or one-step growth experiments.
Burst size	Number of virions released per phage-infected bacterium produced per lytic cycle.
One-step growth	Single round of a phage lytic cycle (typically purely lytic) that is assessed in terms of increases in plaque-forming units over time.
Multistep growth	Sequential occurrence of more than one especially purely lytic cycle as resulting in prolonged phage population growth.
Phage population growth	As used here, refers to multistep growth involving a series of virion adsorption steps that are followed by phage purely lytic cycles.
Secondary adsorption	Distinguishing the first phage to infect a bacterium from subsequently adsorbing (secondary) phages; may induce lysis inhibition.
Lysis inhibition	Extension of purely lytic cycle that occurs in certain phages in response to the secondary adsorption of an already phage-infected bacterium.
Multiplicity of infection	Used to describe ratios of phages—whether added, adsorbed, or infecting—to phage susceptible bacteria; abbreviated as MOI.
Multiplicity	Ratio of phages to bacteria, either at the point of phage addition (MOI_input_) or following phage adsorption to bacteria (MOI_actual_).

## Data Availability

Data for the original contributions presented in this study are included in the file, Supplementary Materials. Further inquiries can be directed to the author.

## Supplementary Materials

The following supporting information can be downloaded at: https://www.mdpi.com/article/10.3390/v17121573/s1.

## Appendix A. Lysis Profile Methods

The lysis profile curves presented in this manuscript represent single biological replicates. Technical replicates have been averaged together to generate the displayed curves unless they are shown separately. Error bars are not provided for averaged technical replicates because such replicates lack statistical independence—they represent repeated measurements from the same biological sample and therefore cannot be used to make statistical inferences. When working with lysis profiles, it is also advisable to inspect each technical replicate individually before averaging, as variations in curve shape may reflect measurement artifacts or biological variation that warrants recognition, since those can otherwise lead to misleading averages.

Experiments unless otherwise indicated were undertaken using a Molecular Devices Thermomax microtiter plate reader set to 37 °C, with 3 s of shaking prior to each time point, taken every four min using a 650 nm filter. The latter wavelength overlaps the “66” Klett-Summerson Photoelectric Colorimeter filter also as used here. In that instrument’s manual, that filter is described as possessing “a spectral range of 640–700 millimicrons”, also known as nanometers, and is a filter used for quantification of bacterial turbidity. Unless otherwise noted, the bacterial host is *E. coli* B and the media used is Muller-Hinton Broth II, cation adjusted.
